# Pap Testing in a High-Income Country with Suboptimal Compliance Levels: A Survey on Acceptance Factors among Sicilian Women

**DOI:** 10.3390/ijerph15091804

**Published:** 2018-08-22

**Authors:** Vincenzo Restivo, Claudio Costantino, Antonello Marras, Giuseppe Napoli, Sabrina Scelfo, Tiziana Scuderi, Alessandra Casuccio, Achille Cernigliaro, Angela Giusti, Stefania Spila Alegiani

**Affiliations:** 1 Department of Science for Health Promotion and Mother-Child Care “G. D’Alessandro”, University of Palermo, Via del Vespro 133, 90127 Palermo, Italy; claudio.costantino01@unipa.it (C.C.); g.napoli15@virgilio.it (G.N.); alessandra.casuccio@unipa.it (A.C.); 2 Department of Health Services and Epidemiological Observatory, Regional Health Authority, Sicilian Region, Via Mario Vaccaro 5, 90145 Palermo, Italy; antonello.marras@regione.sicilia.it (A.M.); achille.cernigliaro@regione.sicilia.it (A.C.); 3 Regional Center for Paediatric Diabetes and Obesity, Azienda Sanitaria Provinciale Caltanissetta, Via Malta 71, 93100 Caltanissetta, Italy; sabrinascelfo@alice.it; 4 Preventive Health Department, Cancer Registry, ASP Trapani, Via Ammiraglio Staiti 95, 91100 Trapani, Italy; trusyit@yahoo.it; 5 National Centre for Epidemiology, Surveillance and Health Promotion, National Institute of Health, Viale Regina Elena 299, 00161 Rome, Italy; angela.giusti@iss.it (A.G.); stefania.spila@iss.it (S.S.A.)

**Keywords:** Pap test, screening, prevention, general practitioner, health belief model, cervical cancer, refusal, susceptibility

## Abstract

Cervical cancer screening is uncommon, especially in low-income countries and among lower socioeconomic status people in high-income countries. The aims of this study were to examine the adherence of Sicilian women to Pap testing and to identify the determinants of this in a population with a secondary prevention attitude lower than high-income countries and the national average. A cross-sectional study called “Save Eva in Sicily” was conducted among all women aged 25–64 years, with a sample drawn by the list of general practitioners (GPs), using a proportional sampling scheme, stratified by age and resident population. The study outcome was performing a Pap test within the past three years. The association between the outcome and Pap test determinants was analyzed through a multivariable logistic regression. Among the 365 interviewed women, 66% (*n* = 243) had a Pap test during the last 3 years. On the other hand, 18% of the other women (*n* = 66) had performed at least one Pap test previously and 16% (*n* = 56) had never had a Pap test. In a multivariable model, GPs’ advice (adjusted OR 2.55; 95% CI 1.57–4.14) and perceived susceptibility (adjusted OR 3.24; 95% CI 1.92–5.48) increased the likelihood of the execution of a Pap test. The “Save Eva in Sicily” study identified GP advice and perceived cancer severity as the main correlates of Pap testing among Sicilian women, producing evidence regarding how policy makers can increase compliance. Interventions to increase Pap test adhesion should focus on stimulating GPs to identify patients who regularly do not undergo it and to recommend testing on a regular basis to their patients.

## 1. Introduction

Cervical cancer is the third most common malignancy the fourth cause of death worldwide among women, with an estimated 527,600 new cases and 265,700 deaths in 2012 [1]. It is the first cancer recognized by the World Health Organization as totally attributable to infection, caused by high oncogenic risk strains of the human papilloma virus (HPV) [2].

In Italy, during the course of a lifetime, one in every 170 women experiences cervical cancer, with a standardized incidence rate (SIR) slightly lower in Southern (6.0 per 100,000) than Northern (6.7 per 100,000) and Central (6.6 per 100,000) Italy [3]. The mortality trend is more heterogeneous, with lower rates in the South than other Italian areas. In Sicily in particular, the SIR is 6.2 per 100,000 women with a mortality rate of 1.13 per 100,000 [4].

Cervical cancer screenings detect precancerous lesions and early-stage diseases, thus decreasing cervical cancer mortality [5,6]. In a meta-analysis of 12 case-control studies, cytology screening was strongly associated with a decreased risk of invasive cervical cancer (OR 0.35, 95% CI 0.30–0.41) [5]. However, this public health practice is uncommon, especially in low-income countries, with the lowest adhesion reported in Bhutan (6%) [7]. In high-income countries with established and free of charge screening programs, there is an increased level of Pap test adherence although people from lower socioeconomic backgrounds have a much lower screening uptake than the national average [8,9]. In Italy, Pap testing was performed free of charge with a coverage in 2017 of at least 80% among resident people [10]. From 2014 to 2016, 79% of women aged 25–64 years performed Pap testing through organized programs or voluntarily, with a clear gradient in coverage from Northern (87%) to Southern (68%) Italy [10].

Health beliefs have an influence on women’s health practices, and the health belief model (HBM) is a useful theoretical basis to explain preventive health behaviors such as the actions taken to prevent, screen for, and control illness [11]. However, only a few studies have assessed the relationship between HBM variables and cervical cancer screening [12,13,14].

Furthermore, the most common reason for non-adherence to organized screening against cervical cancer was the perception that Pap testing was not useful and a lack of information and counseling supplied by general practitioners (GPs) [10,15,16].

The aims of this study were to examine the adherence to Pap testing of Sicilian women and to identify the determinants of this in a population with a secondary prevention attitude lower than high-income countries and the national average.

## 2. Materials and Methods

### 2.1. Study Design

A cross-sectional study called “Salvate Eva in Sicilia” (SES, Save Eva in Sicily) was conducted from January to June 2016 by participants attending the university of Palermo Master’s program “Promozione della Salute della Popolazione e Epidemiologia Applicata alla Promozione” (PROSPECT, Population Health Promotion and Preventive Epidemiology) in the Sicily region (an administrative Southern Italian region with a population of about five million inhabitants, of which 1,395,866 are women aged 25–64 years).

The eligible population included all women aged 25–64 years (age calculated as of 1 January 2016), who are residents in Sicily and are taken care of by the National Health Service. The sample was drawn by the list of general practitioners’ enrollees from all nine Sicilian local health units (LHUs), using a proportional sampling scheme, stratified by the resident population of each LHU.

Exclusion criteria were the unavailability of a telephone number, no response by women after at least three attempts (two attempts in the morning/afternoon and one attempt in the evening), residence or domicile outside the LHU, hysterectomy, refusal to answer the questionnaire, institutionalization (convent, prison, hospital, etc.), inability to talk in Italian, severe mental or physical disabilities and death.

An informative letter, describing the purpose of the SES survey, was sent to all sampled women and their GP. A standardized questionnaire was administered through a telephone interview to investigate the women’s knowledge about cervical cancer as well as their compliance to screening. The interviewers were medical residents in Hygiene and Preventive Medicine at Palermo University, Italy. A common training of all interviewers was performed to standardize the procedures and to ensure the quality of data collection. All women matching the exclusion criteria were replaced with other eligible ones sharing the same characteristics, selected from a list of substitutes obtained with the same sampling procedure. At the beginning of the interview, informed consent was obtained and survey aims were explained as well as methods used to ensure confidentiality of data. At the end of the interview, women received health advice if requested. The study was approved by the ethical committee of Palermo 1 on 11 November 2015 (ID number 10/2015).

Before the beginning of the study, the questionnaire was validated in a convenience sample representing approximately 10% of the women. The collected data were entered in a dedicated database through an online custom-made user interface (using the Survey Monkey software) and a quality control check of data entry was performed before data analysis.

### 2.2. Study Variables

The questionnaire variables were selected according to national and international literature on Pap test adherence and its determinants [10,15,16], and it included the following items: (a) characteristics of the population (age, school level, working activity, cohabitation, marital status, parity); (b) Pap test practice (prevalence of women who performed a preventive Pap test; periodicity, type of healthcare provider and costs); (c) information on Pap testing received by invitation letter and/or a GP’s advice and/or an informative mass campaign; (d) attitudes, beliefs, and opinions on Pap testing and cervical cancer, explored according to the HBM and measured with a five-point Likert scale (perceived susceptibility as risk of developing cancer; perceived severity of the disease and its consequences; perceived benefits related to prevention; perceived barriers to perform Pap testing, only for women who had not performed a Pap test in the last three years). To better characterize the women’s knowledge and attitudes about other preventive measures, the questionnaire reported some additional items on the HPV DNA test, the HPV vaccine, mammography, and fecal occult blood (FOB) testing.

### 2.3. Data Analysis

The sample size (*n* = 439) was estimated using an expected prevalence of screening coverage of 50%, a confidence level of 95%, and an accuracy of ±4.5%.

The study outcome was performing a Pap test within the past three years. Complex survey design analyses using the Taylor series method for variance estimation were conducted in the Stata 11 software (Stata Corporation, College Station, TX, USA). Percentage estimates were weighted by the sampling fraction in each LHU stratum. The association between the outcome and Pap test determinants was analyzed through the chi-square test for categorical variables and Student’s *t*-test for continuous ones. A multivariable logistic regression model was performed, taking into account potential confounding factors. The determinants of Pap testing, in which *p* < 0.25 in univariate analysis, and variables with at least 20 women for each subgroup were included in the multivariable models. Furthermore, age was included in multivariable model as an a priori confounder. Epi Info (Centers for Disease Control, Atlanta, GA, USA) and SPSS software (IBM, Armonk, NY, USA) was used for the statistical analyses.

## 3. Results

**Figure 1:**
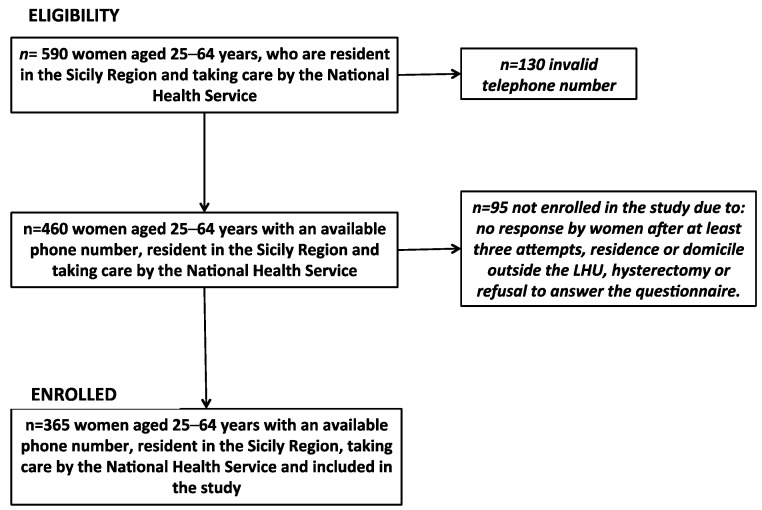
Flow chart of “Save Eva in Sicily” study.

The sample consisted of 590 eligible women aged 25–64 years old (Figure 1). Of these, 130 (22.0%) had an invalid telephone number, making a total of 460 contactable women. Ninety-five of these (20.7%) were excluded from analysis due to not responding after at least three attempts, residing outside Sicily, having had a hysterectomy, or refusal to answer the questionnaire.

The study had a response rate of 79.3% (*n* = 365), and the interviewed women overlapped with the resident female population of the LHU. The enrolled women had a median age of 52 years (IQR 39–59), 46% had a low school level (less than 8 years) and about 80% had at least one childbirth (Table 1).

Fifty-nine percent of women reported a screening invitation letter as the main source of awareness about getting a Pap test, whereas for 52% and 48% of responding women, mass information campaigns and GPs’ advice, respectively, had an important role for awareness. Furthermore, the most relevant source of information on Pap testing (94%) was a healthcare provider (GP, gynecologist, or clinic) followed by friends or the web (6%). According to the HBM, the most important perceptions about Pap testing or cervical cancer were benefits (75%), followed by susceptibility (71%) and severity (67%).

The anti-HPV vaccination and HPV DNA test were known by 248 (68%) and 121 (32%) women, respectively. More than 64% (*n* = 237) of interviewed women reported performing a mammography, while only 21% (*n* = 77) underwent FOB testing once in their life (data not shown).

Among the 365 interviewed women, 66% (95% CI 61–71, *n* = 243) had a Pap test during the last 3 years. On the other hand, 16% (95% CI 12–20, *n* = 56) of women had never performed a Pap test and 18% (95% CI 14–23, *n* = 66) performed at least one Pap test before the last three years.

The most relevant determinants of Pap test adherence (Table 2) were GPs’ advice (OR 2.92; 95% CI 1.83–4.66), perceived benefits of Pap testing (OR 1.94; 95% CI 1.18–3.18) and perceived susceptibility (OR 3.80; 95% CI 2.34–6.18).

The multivariable model considered the effects of age, school level, marital status, invitation letter, cervical cancer source of information, GPs’ advice, perceived susceptibility, severity, and benefits on the likelihood of getting screened. GPs’ advice (adjusted OR 2.55; 95% CI 1.57–4.14) and perceived susceptibility (adjusted OR 3.24; 95% CI 1.92–5.48) increased the likelihood of the execution of Pap testing (Table 2).

**Figure 2:**
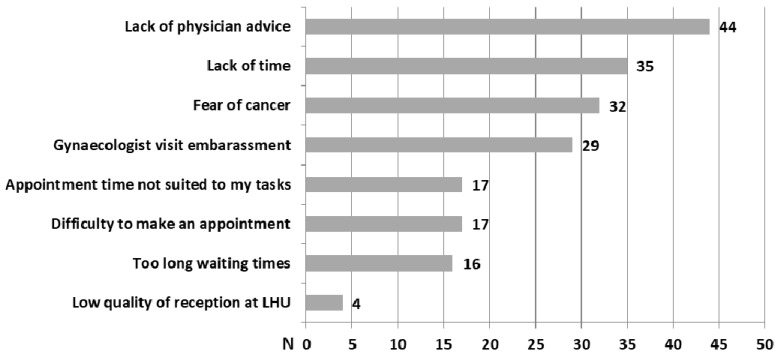
Perceived barriers to Pap test screening for women who did not perform a Pap test during the last three years.

Women who did not perform a Pap test in the last three years most frequently reported as barriers (Figure 2) the lack of physicians’ advice (36%), a lack of time (28%), fear of cervical cancer diagnosis (28%), and embarrassment at visiting a gynecologist (25%).

## 4. Discussion

The SES survey is a study conducted in Sicily on adhesion to Pap testing and its determinants. In Sicily, according to the SES survey, seven out of 10 women performed a Pap test in the last three years based on national guidelines [17]. The prevention of cervical cancer reduces its mortality but, despite this, more than one-half of women who develop cervical cancer have not been screened appropriately [18,19,20,21]. Although these data show a wide dissemination of secondary prevention programs among Sicilian women, the adherence was less than reported in Italy and in other high-income countries [3]. In particular, women who reported taking part in a screening program organized by the LHU (performing screening within the last three years, in a public facility and freely) accounted for 55.8% of sampled women. These figures were far from reaching a level necessary to ensure the effectiveness of an organized screening program [17]. Moreover, almost 34% of Sicilian women did not perform a Pap test during the last three years, while 16% of them had never had one. These women were at higher risk of cancer throughout their lives. This level of screening refusal or delay, similar to low-income countries, could be related to the negative attitude of not undergoing routine health checks without first experiencing health problems [9,22]. Recent studies showed that, in countries with established screening programs, lower socioeconomic groups or people with particular cultural factors have a much lower screening uptake than the national average, even when screening is provided free of charge. Such groups include ethnic minorities, those living in rural areas, and women on low incomes [8,15].

In Italy, there is another survey to monitor preventive services and also cervical screening, called “Progressi delle aziende sanitarie per la salute in Italia” (PASSI), but the SES survey targeted cervical screening with particular detail by investigating association not only with demographic variables as PASSI, but also with variables about perception severity of pathology, susceptibility, barriers, and the source of information on screening adherence [23].

The multivariable analysis shows that the most relevant factor associated to the Pap test was the advice of a GP. Among Sicilian women, the lack of, or ineffective communication with a GP may indicate an absence of influential interactions with health care providers. In fact, more than half of participants in this study reported the lack of a physician’s recommendation; an especially worrisome result since previous findings suggest that a physician’s recommendation is a major significant predictor of screening [24,25]. It will be necessary to focus on GP communication to better understand the quality and content of provider–patient communication about screening [15]. This topic could have a particular value among the Sicilian population due to cultural factors that can play a role for performing Pap testing. This critical point is that there is a need for educational and motivational interventions that encourage healthcare providers, especially those taking care of women with particular cultural characteristics, to promote cervical cancer screening among the vulnerable segments of the population [15,26].

Furthermore, women who reported perceiving the severity of cervical cancer had a significant association with Pap test execution. The ability of the HBM to explain Pap test adherence varies in different populations. Indeed, several studies have reported significant differences among Pap test adherence and the remaining concepts of HBM: perceived benefits, susceptibility, and barriers [12,14]. In the USA, only the knowledge of cervical cancer was associated with its perceived severity [27]. In particular, Sicilian women who perceived that cervical cancer can lead to death or to a hysterectomy and who considered cervical cancer a serious health problem had a greater awareness of the need to perform a Pap test. Further actions should focus on improving the perception of cervical cancer severity among the general population, because women with lower awareness about HPV and cervical cancer did not perceive the seriousness of their potential risk for cervical cancer development [27]. Moreover, the perception of the disease severity was linked to high-quality and timely follow-up in high-income countries [28].

Similar to other studies, the most frequent reported reasons for the non-execution of Pap testing among Sicilians were the lack of physicians’ advice, a lack of time, fear of cancer, and embarrassment at visiting a gynecologist [12,14]. GPs might have a large influence on their patients’ health care decisions and may be effective in addressing a lack of knowledge and cultural barriers, particularly embarrassment, fear of pain, and the relationship between HPV and cervical cancer [15,25]. Moreover, the use of other interventions such as self-sampling or reminders will ensure higher access of women to Pap testing [29].

This study has two main limitations. First, all participants had to have a working telephone to be enrolled in the study and had to declare their consent to be interviewed. Consequently, the reported response rate (79.3%) was reached only after multiple attempts, calling some women multiple times. Nevertheless, participation rates divided by province of residence were representative for the Sicilian resident population.

Furthermore, the execution of Pap testing was self-reported by Sicilian women. Although this may overestimate adherence to Pap testing, the strategy of using a variety of exclusion criteria (such as leaving out institutionalized women, people with severe mental or physical disabilities, and women with a hysterectomy) could allow us to obtain a more accurate adhesion rate.

## 5. Conclusions

The SES study identified GP advice and perceived cancer severity as the main correlates of Pap testing among Sicilian women, producing evidence regarding how policy makers can increase adherence to screening. Interventions should focus on stimulating GPs to identify patients who regularly do not perform Pap testing and to recommend them to regularly complete it.

## Figures and Tables

**Table 1:** Characteristic of enrolled women and difference between adherence to Pap test within the last three years by demographical characteristics, source of information, attitudes, beliefs and opinions on Pap testing and cervical cancer.

Questionnaire Items	All Women *N* = 365	Pap Test within the Last 3 Years *N* = 243 (%)	No Pap test within the Last 3 Years *N* = 122 (%)	*p*
**Age class**				
*≥45*	240	158 (65.0)	82 (67.2)	0.69
*<45*	125	85 (35.0)	40 (32.8)	
**School level**				
*≤8 years*	169	107 (44.0)	62 (50.8)	0.14
*>8 years*	196	136 (56.0)	60 (49.2)	
**Marital status**				
*Not married*	78	47 (19.3)	31 (25.4)	0.19
*Married*	287	196 (80.7)	91 (74.6)	
**Cohabitation**				
*Not alone*	345	227 (93.4)	118 (96.7)	0.25
*Alone*	20	16 (6.6)	4 (3.3)	
**Working activity ^1^**				
*Not employed*	171	110 (45.3)	61 (50.8)	0.31
*Employed*	192	133 (54.7)	59 (49.2)	
**Parity**				
*No*	70	44 (18.1)	26 (21.3)	0.51
*Yes*	295	199 (81.9)	96 (78.7)	
**Awareness to get a Pap test by invitation letter**				
*No*	150	92 (37.9)	58 (47.5)	0.12
*Yes*	215	151 (62.1)	64 (52.5)	
**Awareness to get a Pap test by GP’s advice**				
*No*	188	104 (42.8)	84 (68.9)	<0.01
*Yes*	177	139 (57.2)	38 (31.1)	
**Awareness to get a Pap test by mass information campaign**				
*No*	171	119 (49.0)	52 (42.6)	0.21
*Yes*	194	124 (51.0)	70 (57.4)	
**Cervical cancer source of information ^1^**				
*Friends, web*	24	13 (5.3)	11 (9.1)	0.25
*General pratictioner, gynaecologist, clinic*	340	230 (94.7)	110 (90.9)	
**Perceived benefits**				
*No*	93	51 (21.0)	42 (34.4)	<0.05
*Yes*	272	192 (79.0)	80 (65.6)	
**Perceived severity ^1^**				
*No*	123	76 (31.3)	47 (38.8)	0.13
*Yes*	241	167 (68.7)	74 (61.2)	
**Perceived susceptibility ^1^**				
*No*	108	49 (20.2)	59 (48.8)	<0.01
*Yes*	256	194 (79.8)	62 (51.2)	

^1^ Missing values: n. 2 (Working activity); n. 1 (Cervical cancer source of information, Perceived benefits, Perceived susceptibility).

**Table 2:** Adherence to Pap test within the last three years by characteristics, source of information, attitudes, beliefs and opinions on Pap testing and cervical cancer of sampled women; crude and adjusted logistic regression analysis.

Questionnaire Items	Crude-OR	95% CI	*P*	Adjusted OR ^1^	95% CI	*p*
**Age class**						
*≥45*	1					
*<45*	1.10	0.69–1.77	0.69	1.31	0.78–2.21	0.31
**School level**						
*≤8 years*	1					
*>8 years*	1.40	0.89–2.18	0.14	0.97	0.59–1.58	0.89
**Marital status**						
*Not married*	1					
*Married*	1.42	0.84–2.41	0.19	1.25	0.70–2.24	0.44
**Cohabitation**						
*Not alone*	1					
*Alone*	1.96	0.63–6.09	0.25			
**Working activity**						
*Not employed*	1					
*Employed*	1.27	0.81–1.99	0.31			
**Parity**						
*No*	1					
*Yes*	1.20	0.69–2.09	0.51			
**Awareness to get a Pap test by invitation letter**						
*No*	1					
*Yes*	1.44	0.91–2.26	0.12	1.26	0.77–2.05	0.35
**Awareness to get a Pap test by GP’s advice**						
*No*	1					
*Yes*	2.92	1.83–4.66	<0.01	2.55	1.57–4.14	<0.01
**Awareness to get a Pap test by mass information campaign**						
*No*	1					
*Yes*	0.75	0.48–1.18	0.21			
**Cervical cancer source of information**						
*Friends, web*	1					
*General pratictioner, gynaecologist, clinic*	1.64	0.71–3.81	0.25	1.40	0.5–3.93	0.53
**Perceived benefits**						
*No*	1					
*Yes*	1.94	1.18–3.18	<0.05	1.58	0.89–2.81	0.12
**Perceived severity**						
*No*	1					
*Yes*	1.43	0.9–2.28	0.13	0.94	0.54–1.62	0.82
**Perceived susceptibility**						
*No*	1					
*Yes*	3.80	2.34–6.18	<0.01	3.24	1.92–5.48	<0.01

^1^ Odds ratios (OR, with 95% confidence intervals) are adjusted for age, marital status, school level, invitation letter, GP’s advice, cervical cancer source of information, perceived susceptibility, severity and benefits.
